# Sexual health in adult women with complete androgen insensitivity syndrome: a single centre cross-sectional study

**DOI:** 10.1007/s40618-025-02592-7

**Published:** 2025-04-30

**Authors:** Alessandra Mangone, Eriselda Profka, Giulia Rodari, Claudia Giavoli, Giovanna Mantovani

**Affiliations:** 1 Department of Clinical Sciences and Community Health, Department of Excellence 2023-2027, University of Milan, Milan, Italy; 2 Endocrinology Unit, Fondazione IRCCS Ca’ Granda Ospedale Maggiore Policlinico, Milan, Italy; 3 Endocrine Unit, Department of Clinical Sciences and Community Health, Department of Excellence 2023-2027, Fondazione IRCCS Ca’ Granda–Ospedale Maggiore Policlinico, Via Francesco Sforza, 35, 20122 Milan, Italy

**Keywords:** Complete androgen insensitivity, DSD, Sexual health, Body uneasiness, FSFI

## Abstract

**Objective:**

We aimed to assess sexual health and body uneasiness in patients with complete androgen insensitivity syndrome (CAIS) using validated questionnaires, since literature is limited and heterogeneous.

**Methods:**

Single-center, cross-sectional study on 34 adults with 46, XY karyotype and confirmed androgen receptor mutation (age = 34 ± 8.6 years), 29 gonadectomized and 5 with gonads in situ. All gonadectomized patients but 3 were receiving hormonal replacement therapy (HRT): 14 with transdermal oestradiol, 10 with oral oestradiol, 2 with testosterone. We measured hormonal levels and adherence to HRT, and we administered Female Sexual Function Index (FSFI), Female Sexual Distress Scale-Revised (FSDS-R) and Body Uneasiness Test (BUT) questionnaires.

**Results:**

We registered sexual dysfunction with FSFI score < 26.55 and FSDS-R score > 11 respectively in 77% and 52.9% of the cohort; body uneasiness (BUT-Global Severity Index > 1.2) was also present in 50%. In patients with gonads in situ, body uneasiness and sexual distress were common, with 4/5 pathological scores at FSFI and BUT, and 2/5 at FSDS-R. In gonadectomized patients, no significant differences among type of HRT and questionnaires’ results were registered. Despite receiving HRT and referring optimal compliance, most (69%) patients still had oestradiol concentration < 50 pg/ml, with no significant correlation with sexual function. 10/34 patients (29%) had vaginal hypoplasia and 9 underwent vaginal dilation treatment, with no correlation with sexual scores.

**Conclusions:**

Sexual dysfunction and body uneasiness are worryingly common in CAIS. Preservation of gonads and HRT do not guarantee adequate sexual function nor optimal hormone levels. Psychological and sexual aspects must be considered in the management and therapeutic choices for these patients, as they highly impact quality of life.

**Supplementary Information:**

The online version contains supplementary material available at 10.1007/s40618-025-02592-7.

## Introduction

Sexuality notoriously contributes to psychological health and well-being, and androgens are believed to play an important role in female sexual function [1].

Androgen insensitivity syndrome (AIS) is a major disorder of sex development (DSD), caused by mutations in the Androgen Receptor (AR) gene in patients with XY-karyotype, leading to tissue resistance to androgens even since the intrauterine life [2]. It is an extremely rare condition, with an estimated prevalence of 1:20,000–1:100,000 births [3]. In the complete phenotype (CAIS) individuals present female external genitalia and female psychosexual development, and they are usually diagnosed during puberty due to primary amenorrhoea [3]. Internal genitalia are absent due to the regression of the Müllerian structures (uterus, cervix and proximal vagina), induced by the normal production of anti-Müllerian hormone (AMH) by the testes, which are usually retained in the abdomen or inguinal canal. Meanwhile, complete insensitivity to testosterone prevents the formation of Wolffian structures, leading to the absence of seminal vesicles and prostate. The vagina has a blind bottom, with a length ranging from 2.5 to 8 cm, often causing later difficulties in sexual intercourse.

Malignant transformation of the gonads is one of the most feared complications in CAIS, but the actual oncological risk and the timing of gonadectomy are still open issue of debate. Recent studies have demonstrated the oncological risk in children with CAIS to be very low, hence gonadal surgery is suggested to be delayed until complete pubertal development, permitting spontaneous puberty and an autonomous decision about surgery [4, 5]. After gonadectomy hormone replacement therapy (HRT) is mandatory, usually based on transdermal (gel or patch) or oral oestrogens, but there is no unique protocol nor any evidence-based data on the optimal dose and monitoring parameters of HRT in CAIS. Recent data have proposed testosterone as an alternative HRT, proving it to be well tolerated and safe and demonstrating a positive effect of testosterone in term of sexual desire which was possibly referred to site-specific conversion of testosterone to oestradiol in the brain by aromatase and 5-α-reductase [6], but long-term data are still needed.

A lack of sexual confidence and a reduction in sexual satisfaction have indeed been observed in women with CAIS [7–13], yet in highly heterogeneous cohorts reporting conflicting results, mostly employing not validated and non-objective methods of sexual function assessment. To date, no specific guidelines on how to address and manage these symptoms are available, and long-term psychological and sexual outcomes remain poorly studied.

We therefore aimed to systematically assess sexual health and body uneasiness in adult women with CAIS through validated questionnaires, including patients with gonads in situ and taking into consideration hormonal status and patients’ adherence to therapies, as well as clinical features such as gynecologic anomalies which may have an impact on sexual outcomes.

## Patients and methods

### Study design and patient’s characteristics

We conducted a single-center, cross-sectional study recruiting all adult patients with CAIS referring to our outpatient clinic at the Endocrinology Unit of Fondazione IRCCS Ca’ Granda Ospedale Maggiore Policlinico in Milan, Italy, between 2019 and 2024. Study eligibility criteria included age between 18 and 50 years, 46, XY karyotype with confirmed androgen receptor (AR) mutation and external female genitalia and signed informed consent. Of the 38 adult patients actively followed-up in our Centre, 4 refused to participate to the study, therefore a total 34 patients with CAIS (mean age 34 ± 8.6 years) was included. The local ethics committee approved this protocol study (Milan, approval number 745_2019bis) and all patients provided written informed consent.

At recruitment, 29 patients have had gonadectomy at a mean age of 12.2 ± 8.8 years, whereas 5 had gonads in situ and were maintaining radiological surveillance. At evaluation, when firstly referred to our Centre, all gonadectomized patients were receiving hormonal replacement therapy (HRT) except for 3 patients who refused it for personal reasons. In particular, 14 women were treated with transdermal bioidentical oestradiol (7 with gel formulation with a median dosage of 2 mg/day and 7 with transdermal patch with a median dosage of 100 μg /24 h) and 10 with oral estradiol-valerate (median dosage 2 mg/day). Two patients were treated with testosterone: 1 with transdermal gel 40 mg/24 h and 1 with injective undecanoate testosterone 1000 mg every 12 weeks.

### Psychosexual assessment

All patients were evaluated through the following validated self-administered questionnaires:

- 19-item Female Sexual Function Index (FSFI), which analyses overall levels of sexual function and its primary components: sexual desire, arousal, lubrication, orgasm, pain, and satisfaction. The FSFI is composed by items with answers codified on a 5-point scale ranging from 1 to 5, with higher scores indicating greater levels of sexual functioning for each item. The total score, resulting from the sum of the five domains, ranges from 2 to 36; a total score of 26.55 has been found to provide an excellent cut-off to distinguish women with and without sexual dysfunction [14–16]. We used the validated Italian version of the scale [17]. This questionnaire was administered only to women sexually active within the last 4 weeks; therefore 12 subjects (35.3%) were excluded from this analysis.
- 13-item Female Sexual Distress Scale-Revised (FSDS-R), consisting of 13 items that relate to different aspects of sexually related personal distress. Higher scores indicate more sexual distress with a total score ≥ 11 being indicative for significant distress [18].
- Body Uneasiness Test (BUT), assessing body image concerns, avoiding and compulsive self‐monitoring, experience of depersonalization and dissatisfaction. It comprises questions regarding body experiences (BUT‐A) and dissatisfaction with self-body parts (BUT‐B). Higher scores indicate greater body uneasiness. We analysed the total score of the test (Global Severity Index, BUT‐GSI) [19].

All questionnaires administered are reported in Supplementary Materials. Questionnaire’s results were compared with the respective test validation population as controls.

None of the patient was receiving any psychiatric drug at evaluation.

### Hormonal and clinical assessment

Clinical and hormonal data at the time of sexual health evaluation were recorded including gonadal status, age at gonadectomy, HRT regimen and dosage, age at start of therapy and adherence to prescribed therapy. Adherence to HRT was reported as intermittent in 5 subjects.

We measured weight, height and Body Mass Index (BMI), defined as weight(kg)/height(m2). Our clinical practice also involves a baseline gynecological evaluation to identify potential anatomical anomalies that may cause difficulties in achieving complete sexual intercourse and contribute to feelings of inadequacy. Data on the prevalence of vaginal hypoplasia in the cohort was therefore evaluated, as well as on the type of vaginal dilation treatment undertaken.

Hormonal data were recorded including centralized assessment of follicle-stimulating hormone (FSH), luteinizing hormone (LH), total testosterone and 17 beta-estradiol levels, measured fasting in the morning with highly sensitive radioimmunoassays in our Center at Fondazione IRCCS Ca’ Granda Ospedale Maggiore Policlinico of Milan.

### Statistical analysis

We described continuous parameters with normal distribution as mean ± standard deviation (SD) and non-Gaussian distributions as median with interquartile range (IQR). Continuous and non-Gaussian data were compared using t-test and Wilcoxon–Mann–Whitney test, respectively. Categorical data were presented as percentage (%), proportion (/) and analyzed using the Chi-squared test or Fisher's exact test as appropriate. P-values < 0.05 were considered statistically significant. Statistical analyses were conducted using IBM SPSS 28.0 (IBM Analytics).

## Results

### Characteristics of the study cohort

The demographic and hormonal data of the study cohort are detailed in Table 1. Patients were stratified according to gonadal status in two groups: gonadectomized (n = 29) and with gonads (n = 5). The two groups differed in FSH and testosterone level at evaluation (p = 0.03 and p < 0.01, respectively), as expected, and in term of body mass index (BMI), with patients with gonads presenting higher BMI at evaluation (median 32.8 kg/m2 vs 21.4 kg/m2, p = 0.02).

**Table 1:** Demographic and hormonal data of the study cohort

Parameter	Total cohort	Gonadectomized	With gonads in situ	P value
Demographic	(n = 34)	(n = 29)	(n = 5)	
Age, years	34 (27–39)	35.8 (29–39)	27.5 (25–29)	0.21
BMI, kg/m2	22 (19–27)	21.4 (19–24)	32.8 (31–33)	**0.02**
Hormonal values				
LH, mU/L	35.7 (27–49)	37.1 (28–49)	27.5 (27–29)	0.41
FSH, mU/L	64.9 (27–76)	68.8 (33–76)	8.6 (5.6–10)	**0.03**
Estradiol, pg/mL	33.7 (13–48)	33.6 (7.5–72)	34.7 (26–41)	0.88
Testosterone, ng/ml	0.2 (0.09–0.4)	0.2 (0.09–0.31)	3.1 (2.9–4.4)	** < 0.01**

Bold font indicates a statically significant p-value

Data are expressed as median (interquartile range—IQR) as per non-Gaussian distribution

### Psychosexual assessment

Median score results of each questionnaire are reported in Table 2, showing results in all evaluated psychosexual parameters, with no significant differences among gonadectomized subjects and patients with gonads.

**Table 2:** Questionnaire results of the study cohort and respective pathological score for interpretation

	Total cohort	Gonadectomized	With gonads in situ	P value	Pathological score
FSFI	25.7 (21.2–29.4)	26 (21.4–29.7)	24.5 (20–26)	0.27	< 26.55
FSDS-R	18 (8–29)	18.5 (8–29)	11 (4–21)	0.48	≥ 11
BUT-GSI	1.2 (0.6–1.9)	1.08 (0.6–2.1)	1.4 (1.2–1.8)	0.44	> 1.2

Data are expressed as median (interquartile range—IQR) as per non-Gaussian distribution

Legend: FSFI: Female Sexual Function Index; FSDS-R: Female Sexual Distress Scale-Revised (FSDS-R); BUT-GSI: Body Uneasiness Test-Global Severity Index

**Fig. 1:**
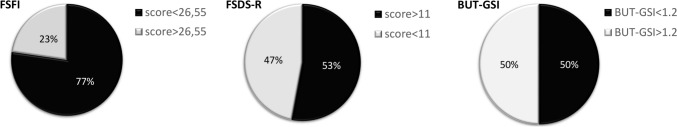
Questionnaires’ results in the study cohort with respective percentage of patients with pathological scores. Legend: FSFI: Female Sexual Function Index; FSDS-R: Female Sexual Distress Scale-Revised (FSDS-R); BUT-GSI: Body Uneasiness Test-Global Severity Index

We registered sexual dysfunction (FSFI score < 26.55) in 77% of patients and sexual distress (FSDS-R ≥ 11) in 52.9% of the cohort. Moreover, 17/34 patients (50%) reported body uneasiness with a BUT-GSI > 1.2. Data and percentages are detailed in Fig. 1.

Interestingly, when focusing on the subgroup with gonads in situ, body uneasiness and sexual dysfunction resulted extremely high, with 4/5 patients reporting a pathological score at FSFI and GSI-BUT, and 2/5 also at FSDS-R scale.

**Fig. 2:**
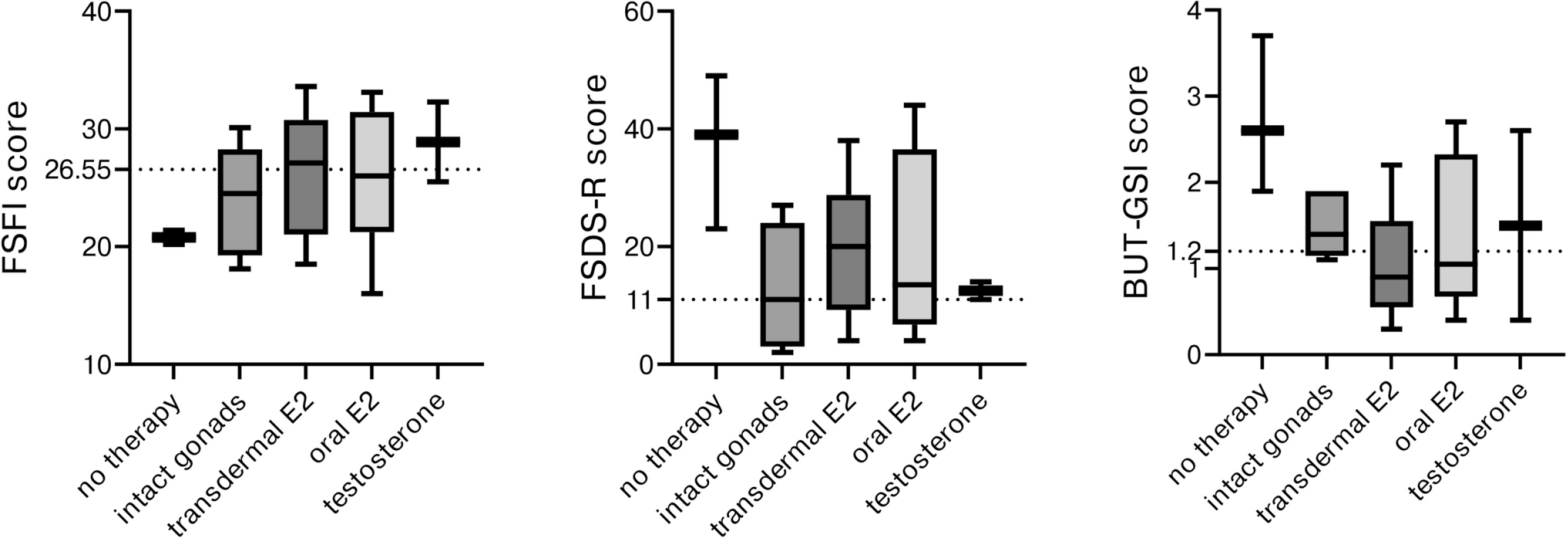
Questionnaires’ results according to gonadal status and type of hormonal replacement therapy received at evaluation. Data are express as median, range and IQR. Legend: FSFI: Female Sexual Function Index; FSDS-R: Female Sexual Distress Scale-Revised (FSDS-R); BUT-GSI: Body Uneasiness Test-Global Severity Index. Dotted lines represent the cut-off for respective pathological score: FSFI < 26.55; FSDS-R ≥ 11; BUT-GSI > 1.2

In gonadectomized patients, no significant differences among different HRT regimes and questionnaires’ results were registered. Results of sexual questionnaires and their distribution according to gonadal status and type of HRT received are reported in Fig. 2.

The patients who were not taking HRT for compliance reasons showed pathological results in every questionnaire, with median FSFI score of 20.8 (range 20.2–21.4), median FSDS-R of 39 (23–49) and median BUT-GSI of 2.6 (19–3.7). Of the two patients with testosterone therapy, one had normal scores.

We additionally performed a sub analysis of the six subscales of FSFI questionnaire, confirming the overall impairment with no specific differences with respects to the gonadal status (see Supplementary Table 1) nor among the different HRT regimes (data not shown).

### Hormonal and clinical assessment

**Fig. 3:**
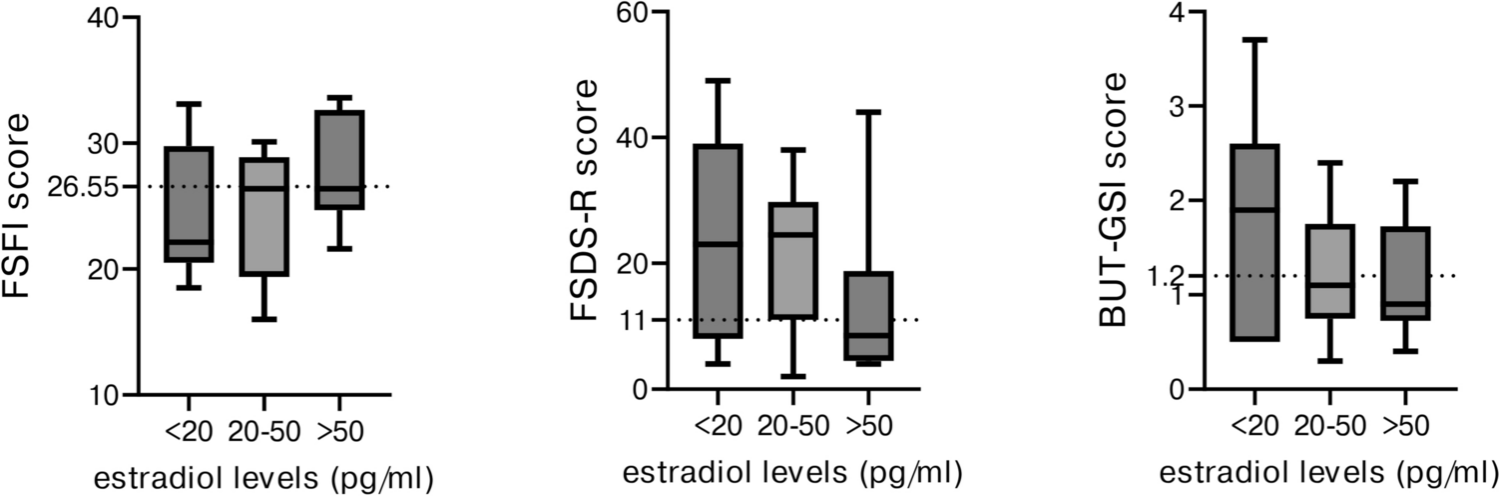
Questionnaires’ results according to oestradiol levels (pg/ml) at evaluation. Data are express as median, range and IQR. Legend: FSFI: Female Sexual Function Index; FSDS-R: Female Sexual Distress Scale-Revised (FSDS-R); BUT-GSI: Body Uneasiness Test-Global Severity Index. Dotted lines represent the cut-off for respective pathological score: FSFI < 26.55; FSDS-R ≥ 11; BUT-GSI > 1.2

Despite receiving standard HRT-dose and mostly referring optimal compliance, most (69%) patients had serum oestradiol concentration < 50 pg/ml, with median oestradiol levels in gonadectomized patients of only 33.6 pg/ml (IQR 7.5–72), also excluding the 5 patients who reported suboptimal compliance to therapy (median oestradiol 36.8 pg/ml). A slight trend between oestradiol levels and better sexual function was observed, with patients with oestradiol levels > 50 pg/ml reporting median result at all questionnaires within the normal ranges (as detailed in Fig. 3), yet without reaching statistical significance.

Vaginal hypoplasia was present in 10/34 patients (29%), of whom 9 had underwent vaginal dilation treatment, which was performed in 3 subjects through laparoscopic modified Davydov vaginoplasty and in 6 women through nonsurgical self-vaginal dilation. No correlations between presence of vaginal hypoplasia, vaginal dilation treatment and the three sexual questionnaires were registered.

## Discussion

In this study on a large monocentric cohort of women with genetically defined CAIS syndrome, sexual health impairment and body uneasiness resulted worryingly common in adult patients.

Sexual dysfunction has already been reported in CAIS, although findings vary across highly heterogeneous cohorts. In particular, Minto et al. [10] investigated sexual function in a relatively large group of patients with CAIS through the Golombok-Rust Inventory of Sexual Satisfaction scale and found a high prevalence of sexual difficulties, especially sexual infrequency and vaginal penetration difficulty. These results contrast with earlier studies, mostly reporting satisfactory sexual outcomes in CAIS, yet in small series often employing non-validated methods of sexual function assessment [7]–[9]. More recently, Fliegner et al. [11] examined sexual health in 11 patients with CAIS, all gonadectomized, and reported a lack of sexual confidence and satisfaction, excluding clinically relevant depression as a confounding factor. FSFI results were available in only 6 patients, as the rest were not sexually active [11]. The dsd-LIFE multicentre study included 69 individuals with CAIS, analyzed together with complete XY gonadal dysgenesis, XX gonadal dysgenesis and unknown steroid synthesis defects (XY-DSD + XX-GD-F–na group) and found this group to be least satisfied with sex life compared to individuals with other DSD conditions, with over 50% reporting lack of sexual desire when asked [12]. Conversely, in the study by Engberg et al. 20 women with CAIS were assessed showing normal sexual functioning using the Profile of Female Sexual Function and Personal Distress Scale scales, comparable to population-derived controls and women with POI [13]. Thus, available data on sexual function in patients with CAIS remain inconclusive, with significant variability in both the cohorts studied and the methods of assessment used. Moreover, the ongoing HRT is not specified in previous literature on the subject.

Beyond sexual dysfunction, we believe that our result on the highly reported distress related to sexual life registered trough the FSDS-R scale is also concerning, since it clearly influences well-being.

Interestingly, in the present study sexual impairment resulted highly prevalent also in the subgroup of patients with gonads in situ. Despite this subset of patients being very small, fully reflecting clinical practice, we believe it is an interesting preliminary result, also considering that this is the first study to investigate this issue. In fact, avoiding gonadectomy and maintaining the gonads has been suggested by some authors to help maintaining sexual health: in our experience, the presence of functional gonads did not seem to preserve sexual well-being.

We also systematically assess the aspect of body uneasiness, on which very limited data are available. Wisniewski et al. [7] previously assessed body image perception in a sample of 14 subjects with CAIS, using a questionnaire with three response options (mainly satisfied, somewhat dissatisfied, mainly dissatisfied) and also reported varying degree of body dissatisfaction in 43% of the cohort, related to physical appearance and linked to the presence of obesity. In the dsd-LIFE study [20], participants with 46,XY conditions showed different results concerning body image, as expected given the heterogeneous group composition, with female-identifying participants registering more positive scores than participants with partial androgen action or hypospadias, but no specific data on CAIS are available.

In our study, the patients who were not taking HRT performed worse in all questionnaires, which could be expected and possible biased by a worse attitude toward the psychological and medical issues. We did not register significant differences among different HRT regimes and questionnaires’ results, but this could have been influenced by the small number of patients included in each subgroup, and further multicentric studies are needed to better assess this aspect. As mentioned, very limited data are available in CAIS; however, similar results have been observed in young women with spontaneous POI, where FSFI scores resulted lower in comparison to age-matched controls despite taking HRT with optimal compliance, suggesting other factors playing a role in sexuality outcomes [21].

The FSFI questionnaire was also used in the recent study by Birnbau et al. [6] to explore the differences in sexual function between testosterone and oestradiol transdermal therapy: at the end of the study, the difference of FSFI total score between the two groups was not significant, and testosterone resulted superior to oestradiol only in improving the domain of sexual desire [6]. In the present study we could not properly explore this point, as at recruitment only 2 of our patients were receiving testosterone, yet we still noticed that their sexual and arousal domains’ results were in the high-normal range. It will be interesting to further explore the different sub-domains of sexual health in larger cohorts. Considering the high prevalence of sexual dysfunction in our patients, a trial with testosterone therapy aiming to improve sexual desire could be worth a try in the future, even though the unsatisfactory results registered with respect to the overall sexual satisfaction in the mentioned trial [6] seems to suggest it might not be enough.

Another issue that probably needs to be better explored in the future is target oestradiol levels and the goals of HRT. Since no specific guidelines on the optimal management are available for patients with CAIS, we derived target oestradiol levels during HRT from the most recent European guidelines for POI, suggesting to aim to obtain physiological oestradiol levels as found in the serum of women with normal menstrual cycles, average 50–100 pg/ml [22]. The recent guidelines on Turner syndrome propose even higher goals of oestradiol concentrations of 100–150 pg/ml [23]. Our study underlines how in clinical practice these levels seem to be hard to achieve: as shown, most of our cohort had insufficient serum oestradiol concentration < 50 pg/ml, and none of them levels above 100, therefore we could not explore if higher oestrogen levels could have resulted in improved sexual health. The type of formulation employed and the distance from the last administration could also impact the oestradiol levels registered.

Finally, in our study the prevalence of sexual dysfunction resulted high regardless of the presence of vaginal hypoplasia and of the dilation treatment performed. Again, this observation could be affected by the small cohort, and it is consistent with previous limited literature. In the study by Minto et al. mentioned above [10], women with shorter vaginal length had a higher incidence of vaginal penetration problems, yet without reaching statistical significance. Previous dilator treatment for vaginal hypoplasia did not affect sexual function scores nor self-perceptions of vaginal normality [10]. Further multicentric studies are needed to better explore the impact of vaginal hypoplasia and the efficacy of treatment on the incidence of sexual difficulty. In this regard, it is worth noticing how, as underlined in the review by Callens et al. [24], the definition itself of functional success after treatments for vaginal hypoplasia is quite variable and mostly defined as “satisfactory sexual intercourse” without relying on standardized methods of assessment. Only a few studies have utilized reliable questionnaires (i.e. FSFI), and none focusing specifically on CAIS [24].

In patients with CAIS, the burden of sexual distress is complex and multifaceted and could be referred not only to the effect of the physical and hormonal changes, but also to the emotional burden of the diagnosis and its consequent infertility, which can have consequences on self-perception and relationships. Moreover, age at evaluation and cultural and economic conditions can also play a role.

The main limitation of the present study is the small number of patients included. However, the rarity of the condition and the lack of centralized adult care pose challenges in collecting data on patients with CAIS. Moreover, the cross-sectional design of the work prevents us from drawing conclusions on causal effects. Still, we believe that the present study represents a promising starting point for future multicentre prospective studies to focus on this often-neglected issue. It also presents the strength of stratifying the sexual outcomes analysed according to gonadal status and type of HRT, posing new challenges in the management of the condition. Another limitation is that steroid hormone determination was based on radioimmunoassay and not tandem mass spectrometry. Finally, we did not register the relationship status of the patients included, which also has an impact on sexual results.

In conclusion, our study demonstrates how preservation of gonads and standard-dose HRT does not guarantee adequate sexual function nor optimal hormone levels. Psychological and sexual aspects must be taken into consideration in the management and therapeutic choices for these patients, as they highly impact the quality of life despite being still largely ignored in routine clinical management.

## Supplementary Information

Below is the link to the electronic supplementary material.

Supplementary file1 (PDF 71 kb)

Supplementary file2 (PDF 234 kb)

Supplementary file3 (PDF 67 kb)

Supplementary file4 (DOCX 15 kb)

## Data Availability

The raw data supporting the conclusions of this article will be made available by the authors upon request, without undue reservation.
