# Supplementary material for: Sexual health in adult women with complete androgen insensitivity syndrome: a single centre cross-sectional study
Source: J Endocrinol Invest. 2025 Apr 30;48(8):1849–55. doi: 10.1007/s40618-025-02592-7 (PMC12313786; doi:10.1007/s40618-025-02592-7)
Supplement: Supplementary file 1 — Supplementary file1 (PDF 71 kb) [file 40618_2025_2592_MOESM1_ESM.pdf]

COMPILA QUESTA ULTIMA PARTE DEL QUESTIONARIO SOLO SE SEI DONNA

INDICE DELLA FUNZIONE SESSUALE FEMMINILE (FSFI)

ISTRUZIONI :

Queste domande riguardano i suoi sentimenti sessuali e le relative risposte delle ultime 4 settimane. Le chiediamo di rispondere alle domande nel modo più onesto e chiaro possibile. Le sue risposte saranno considerate confidenziali e custodite nell'assoluta riservatezza. Nel rispondere alle domande la preghiamo di tenere a mente le seguenti definizioni:

Attività sessuale: include l'accarezzamento, i preliminari, la masturbazione ed il rapporto sessuale.

Rapporto sessuale indica la penetrazione del pene in vagina.

Stimolazione sessuale comprende situazioni come i preliminari con il partner, l'auto-stimolazione (masturbazione) e le fantasie sessuali.

CONTRASSEGNARE UNA SOLA CASELLA PER OGNI DOMANDA

Il desiderio o interesse sessuale è un sentimento che include il desiderio di avere un esperienza sessuale, sentirsi recettivi ai preliminari sessuali del partner e pensare o fantasticare sul fare sesso.

1. Nelle ultime 4 settimane, con quale **frequenza** ha provato desiderio o interesse sessuale?

    ☐ Sempre o quasi sempre
    ☐ La maggior parte delle volte (più della metà delle volte)
    ☐ Qualche volta (circa la metà delle volte)
    ☐ Poche volte (meno della metà delle volte)
    ☐ Mai o quasi mai

2. Nelle ultime 4 settimane, come valuterebbe il suo **livello** (grado) di desiderio o interesse sessuale?

    ☐ Molto alto
    ☐ Alto
    ☐ Moderato
    ☐ Basso
    ☐ Molto basso o assente

L'eccitamento sessuale è una sensazione che comprende sia gli aspetti fisici che mentali. Può includere sensazioni di calore o formicolio nei genitali, lubrificazione (sentirsi bagnata), o contrazioni muscolari.

3. Nelle ultime 4 settimane, con quale **frequenza** si è sentita eccitata (“su di giri”) sessualmente durante l’attività o rapporto sessuale?

   - ☐ Nessuna attività sessuale
   - ☐ Sempre o quasi sempre
   - ☐ La maggior parte delle volte (più della metà delle volte)
   - ☐ Qualche volta (circa la metà delle volte)
   - ☐ Poche volte (meno della metà delle volte)
   - ☐ Mai o quasi mai

4. Nelle ultime 4 settimane, come valuterebbe il suo **livello** di eccitamento sessuale (sentirsi “su di giri”) durante l’attività o rapporto sessuale?

   - ☐ Nessuna attività sessuale
   - ☐ Molto alto
   - ☐ Alto
   - ☐ Moderato
   - ☐ Basso
   - ☐ Molto basso o assente

5. Nelle ultime 4 settimane quanto si è sentita **sicura** del suo eccitamento durante l’attività o rapporto sessuale?

   - ☐ Nessuna attività sessuale
   - ☐ Molto sicura
   - ☐ Abbastanza sicura
   - ☐ Moderatamente sicura
   - ☐ Poco sicura
   - ☐ Pochissimo o per niente sicura

6. Nelle ultime 4 settimane, con quale **frequenza** si è sentita soddisfatta del suo eccitamento durante l’attività o rapporto sessuale?

   - ☐ Nessuna attività sessuale
   - ☐ Sempre o quasi sempre
   - ☐ La maggior parte delle volte (più della metà delle volte)
   - ☐ Qualche volta (circa la metà delle volte)
   - ☐ Poche volte (meno della metà delle volte)
   - ☐ Mai o quasi mai

7. Nelle ultime 4 settimane, con quale **frequenza** si è lubrificata (bagnata) durante l’attività o rapporto sessuale?

   - ☐ Nessuna attività sessuale
   - ☐ Sempre o quasi sempre
   - ☐ La maggior parte delle volte (più della metà delle volte)
   - ☐ Qualche volta (circa la metà delle volte)

☐ Poche volte (meno della metà delle volte)
☐ Mai o quasi mai

8. Nelle ultime 4 settimane, quanto è stato **difficile** lubrificarsi (bagnarsi) durante l'attività o rapporto sessuale?

   ☐ Nessuna attività sessuale
   ☐ Estremamente difficile o impossibile
   ☐ Molto difficile
   ☐ Difficile
   ☐ Moderatamente difficile
   ☐ Nessuna difficoltà

9. Nelle ultime 4 settimane, con quale frequenza è riuscita a **mantenere** la sua lubrificazione (sentirsi bagnata) fino al completamento dell'attività o rapporto sessuale?

   ☐ Nessuna attività sessuale
   ☐ Sempre o quasi sempre
   ☐ La maggior parte delle volte (più della metà delle volte)
   ☐ Qualche volta (circa la metà delle volte)
   ☐ Poche volte (meno della metà delle volte)
   ☐ Mai o quasi mai

10. Nelle ultime 4 settimane, quanto è stato **difficile** mantenere la sua lubrificazione (sentirsi bagnata) fino al completamento dell'attività o rapporto sessuale?

    ☐ Nessuna attività sessuale
    ☐ Estremamente difficile o impossibile
    ☐ Molto difficile
    ☐ Difficile
    ☐ Moderatamente difficile
    ☐ Nessuna difficoltà

11. Nelle ultime 4 settimane, durante la stimolazione o rapporto sessuale, con quale **frequenza** ha raggiunto l'orgasmo?

    ☐ Nessuna attività sessuale
    ☐ Sempre o quasi sempre
    ☐ La maggior parte delle volte (più della metà delle volte)
    ☐ Qualche volta (circa la metà delle volte)
    ☐ Poche volte (meno della metà delle volte)
    ☐ Mai o quasi mai

12. Nelle ultime 4 settimane, durante la stimolazione o rapporto sessuale, quanto è stato **difficile** per lei raggiungere l'orgasmo?

    ☐ Nessuna attività sessuale

☐ Estremamente difficile o impossibile
☐ Molto difficile
☐ Difficile
☐ Moderatamente difficile
☐ Nessuna difficoltà

13. Nelle ultime 4 settimane, quanto si è sentita **soddisfatta** della sua capacità di raggiungere l'orgasmo durante l'attività o rapporto sessuale?

☐ Nessuna attività sessuale
☐ Molto soddisfatta
☐ Moderatamente soddisfatta
☐ Soddisfatta e insoddisfatta in ugual misura
☐ Moderatamente insoddisfatta
☐ Molto insoddisfatta

14. Nelle ultime 4 settimane, quanto è stata **soddisfatta** del contatto emotivo con il suo partner durante l'attività sessuale?

☐ Nessuna attività sessuale
☐ Molto soddisfatta
☐ Moderatamente soddisfatta
☐ Soddisfatta e insoddisfatta in ugual misura
☐ Moderatamente insoddisfatta
☐ Molto insoddisfatta

15. Nelle ultime 4 settimane, quanto è stata **soddisfatta** della relazione sessuale con il suo partner?

☐ Nessuna attività sessuale
☐ Molto soddisfatta
☐ Moderatamente soddisfatta
☐ Soddisfatta e insoddisfatta in ugual misura
☐ Moderatamente insoddisfatta
☐ Molto insoddisfatta

16. Nelle ultime 4 settimane, quanto è stata complessivamente **soddisfatta** della sua vita sessuale ?

☐ Nessuna attività sessuale
☐ Molto soddisfatta
☐ Moderatamente soddisfatta
☐ Soddisfatta e insoddisfatta in ugual misura
☐ Moderatamente insoddisfatta
☐ Molto insoddisfatta

17. Nelle ultime 4 settimane, con quale **frequenza** ha sentito dolore o fastidio durante la penetrazione vaginale?

- ☐ Non ho avuto rapporti
- ☐ Sempre o quasi sempre
- ☐ La maggior parte delle volte (più della metà delle volte)
- ☐ Qualche volta (circa la metà delle volte)
- ☐ Poche volte (meno della metà delle volte)
- ☐ Mai o quasi mai

18. Nelle ultime 4 settimane, con quale **frequenza** ha sentito dolore o fastidio dopo la penetrazione vaginale?

- ☐ Non ho avuto rapporti
- ☐ Sempre o quasi sempre
- ☐ La maggior parte delle volte (più della metà delle volte)
- ☐ Qualche volta (circa la metà delle volte)
- ☐ Poche volte (meno della metà delle volte)
- ☐ Mai o quasi mai

19. Nelle ultime 4 settimane, come valuterebbe il suo **livello** (grado) di fastidio o dolore durante o dopo la penetrazione vaginale?

- ☐ Non ho avuto rapporti
- ☐ Molto alto
- ☐ Alto
- ☐ Moderato
- ☐ Basso
- ☐ Molto basso o assente

Grazie per aver completato il questionario
