# Supplementary material for: Sexual health in adult women with complete androgen insensitivity syndrome: a single centre cross-sectional study
Source: J Endocrinol Invest. 2025 Apr 30;48(8):1849–55. doi: 10.1007/s40618-025-02592-7 (PMC12313786; doi:10.1007/s40618-025-02592-7)
Supplement: Supplementary file 2 — Supplementary file2 (PDF 234 kb) [file 40618_2025_2592_MOESM2_ESM.pdf]

FEMALE SEXUAL DISTRESS SCALE REVISED

In fondo alla pagina si trova un elenco di sentimenti e di problemi che a volte interessano la vita sessuale delle donne.
Leggere attentamente ogni frase e indicare il numero che descrive meglio QUANTE VOLTE NEGLI ULTIMI 30 GIORNI, OGGI COMPRESO, QUEL PROBLEMA LE ABBIA PROVOCATO FASTIDIO O ANSIA.
E' permesso *cerchiare un solo numero*; nel caso in cui si debba cambiare risposta, cancellare attentamente e rispondere a tutte le domande.
Leggere bene l'esempio prima di incominciare, e chiedere chiarimenti se necessario.

Esempio: Quante volte hai provato una **responsabilità personale per i tuoi problemi sessuali?**

MAI	RARAMENTE	A VOLTE	FREQUENTEMENTE	SEMPRE
0	1	2	3	4

Quante volte ti sei sentita ....

1. Angosciata per la tua vita sessuale	0	1	2	3	4
2. Infelice riguardo la tua relazione sessuale	0	1	2	3	4
3. In colpa per le tue difficoltà sessuali	0	1	2	3	4
4. Frustrata per i tuoi problemi sessuali	0	1	2	3	4
5. Ansiosa riguardo al sesso	0	1	2	3	4
6. Inferiore a causa di problemi sessuali	0	1	2	3	4
7. Preoccupata per il sesso	0	1	2	3	4
8. Inadeguata sessualmente	0	1	2	3	4
9. Con rimpianti per la tua sessualità	0	1	2	3	4
10.Imbarazzata riguardo ai tuoi problemi sessuali	0	1	2	3	4
11.Insoddisfatta riguardo la tua vita sessuale	0	1	2	3	4
12.Arrabbiata per la tua vita sessuale	0	1	2	3	4
13. Infastidita per il poco desiderio sessuale	0	1	2	3	4
