# Supplementary material for: Sexual health in adult women with complete androgen insensitivity syndrome: a single centre cross-sectional study
Source: J Endocrinol Invest. 2025 Apr 30;48(8):1849–55. doi: 10.1007/s40618-025-02592-7 (PMC12313786; doi:10.1007/s40618-025-02592-7)
Supplement: Supplementary file 3 — Supplementary file3 (PDF 67 kb) [file 40618_2025_2592_MOESM3_ESM.pdf]

Body Uneasyness Test - BUT A

	MAI	RARAMENT	QUALCHE	SPESSO	MOLTO	SEMPRE
1 Trascorro molto tempo davanti allo specchio	0	1	2	3	4	5
2 Non mi fido del mio aspetto: temo che cambi all'improvviso	0	1	2	3	4	5
3 Mi piacciono gli abiti che nascondono le forme del mio corpo	0	1	2	3	4	5
4 Passo molto tempo pensando a certi difetti della mia immagine fisica	0	1	2	3	4	5
5 Quando mi spoglio evito di guardarmi	0	1	2	3	4	5
6 Penso che la mia vita cambierebbe profondamente se potessi correggere alcuni miei difetti estetici	0	1	2	3	4	5
7 Mangiare in presenza di altri mi provoca ansia	0	1	2	3	4	5
8 Il pensiero di alcuni difetti del mio corpo mi tormenta tanto da impedirmi di stare con gli altri	0	1	2	3	4	5
9 Ho il terrore di ingrassare	0	1	2	3	4	5
10 Faccio lunghi confronti fra il mio aspetto e quello degli altri	0	1	2	3	4	5
11 Se comincio a guardarmi mi è difficile smettere	0	1	2	3	4	5
12 Farei qualsiasi cosa per modificare alcune parti del mio corpo	0	1	2	3	4	5
13 Resto in casa ed evito di farmi vedere dagli altri	0	1	2	3	4	5
14 Mi vergogno dei bisogni fisici del mio corpo	0	1	2	3	4	5
15 Mi sento derisa/o per il mio aspetto	0	1	2	3	4	5
16 Il pensiero di alcuni difetti del mio corpo mi tormenta tanto da impedirmi di studiare o lavorare	0	1	2	3	4	5
17 Cerco nello specchio un'immagine di me che mi soddisfi e continuo a scrutarmi finché non sono sicuro di averla trovata	0	1	2	3	4	5
18 Mi sento più grassa/o di quello che dicono gli altri	0	1	2	3	4	5
19 Evito gli specchi	0	1	2	3	4	5
20 Ho l'impressione che la mia immagine cambi continuamente	0	1	2	3	4	5
21 Vorrei avere un corpo secco e duro	0	1	2	3	4	5
22 Sono insoddisfatta/o del mio aspetto	0	1	2	3	4	5
23 Il mio aspetto fisico è deludente rispetto alla mia immagine ideale	0	1	2	3	4	5
24 Vorrei sottopormi a qualche intervento di chirurgia estetica	0	1	2	3	4	5
25 Non sopporto l'idea di vivere con l'aspetto che ho	0	1	2	3	4	5
26 Mi guardo allo specchio e provo un senso di inquietudine e estraneità	0	1	2	3	4	5
27 Temo che il mio corpo cambi contro la mia volontà in modi che non mi piacciono	0	1	2	3	4	5
28 Mi sento scollata/o dal mio corpo	0	1	2	3	4	5
29 Ho la sensazione che il mio corpo non mi appartenga	0	1	2	3	4	5
30 Il pensiero di alcuni difetti del mio corpo mi tormenta tanto da impedirmi di avere una vita sessuale	0	1	2	3	4	5
31 Mi osservo in quello che faccio e mi chiedo come appaio agli altri	0	1	2	3	4	5
32 Vorrei decidere io che aspetto avere	0	1	2	3	4	5
33 Mi sento diversa/o da come dicono gli altri	0	1	2	3	4	5
34 Mi vergogno del mio corpo	0	1	2	3	4	5

Body Uneasyness Test - BUT B

Del mio corpo in particolare detesto:	MAI	RARAMENT	QUALCHE	SPESSO	MOLTO	SEMPRE
1. La statura	0	1	2	3	4	5
2. La forma della testa	0	1	2	3	4	5
3. La forma del viso	0	1	2	3	4	5
4. La pelle	0	1	2	3	4	5
5. I capelli	0	1	2	3	4	5
6. La fronte	0	1	2	3	4	5
7. Le sopracciglia	0	1	2	3	4	5
8. Gli occhi	0	1	2	3	4	5
9. Il naso	0	1	2	3	4	5
10. Le labbra	0	1	2	3	4	5
11. La bocca	0	1	2	3	4	5
12. I denti	0	1	2	3	4	5
13. Le orecchie	0	1	2	3	4	5
14. Il collo	0	1	2	3	4	5
15. Il mento	0	1	2	3	4	5
16. I baffi	0	1	2	3	4	5
17. La barba	0	1	2	3	4	5
18. I peli	0	1	2	3	4	5
19. Le spalle	0	1	2	3	4	5
20. Le braccia	0	1	2	3	4	5
21. Le mani	0	1	2	3	4	5
22. Il torace	0	1	2	3	4	5
23. Le mammelle	0	1	2	3	4	5
24. Lo stomaco	0	1	2	3	4	5
25. Il ventre	0	1	2	3	4	5
26. I genitali	0	1	2	3	4	5
27. Le natiche	0	1	2	3	4	5
28. Le anche	0	1	2	3	4	5
29. Le cosce	0	1	2	3	4	5
30. Le ginocchia	0	1	2	3	4	5
31. Le gambe	0	1	2	3	4	5
32. Le caviglie	0	1	2	3	4	5
33. I piedi	0	1	2	3	4	5
34. L'odore	0	1	2	3	4	5
35. I rumori	0	1	2	3	4	5
36. Sudare	0	1	2	3	4	5
37. Arrossire	0	1	2	3	4	5
