# Supplementary material for: Sexual health in adult women with complete androgen insensitivity syndrome: a single centre cross-sectional study
Source: J Endocrinol Invest. 2025 Apr 30;48(8):1849–55. doi: 10.1007/s40618-025-02592-7 (PMC12313786; doi:10.1007/s40618-025-02592-7)
Supplement: Supplementary file 4 — Supplementary file4 (DOCX 15 kb) [file 40618_2025_2592_MOESM4_ESM.docx]

**Supplementary Table 1.** Sub analysis of the six domains explored within the Female Sexual Function Index (FSFI) with respect to gonadal status.

|  | **Mean score** | **95% CI lower bound** | **95% CI upper bound** | **p value** |
| --- | --- | --- | --- | --- |
| **FSFI total score** |  |  |  |  |
| Intact gonads | 23,90 |  |  |  |
| Gonadectomized | 25,63 |  |  |  |
| Difference | -1,72 | -6,99 | 3,54 | 0,50 |
| **Desire** |  |  |  |  |
| Intact gonads | 3,96 |  |  |  |
| Gonadectomized | 3,78 |  |  |  |
| Difference | 0,17 | -0,89 | 1,24 | 0,73 |
| **Arousal** |  |  |  |  |
| Intact gonads | 3,84 |  |  |  |
| Gonadectomized | 4,27 |  |  |  |
| Difference | -0,44 | -1,64 | 0,77 | 0,46 |
| **Lubrification** |  |  |  |  |
| Intact gonads | 4,98 |  |  |  |
| Gonadectomized | 4,46 |  |  |  |
| Difference | 0,52 | -0,69 | 1,71 | 0,38 |
| **Orgasms** |  |  |  |  |
| Intact gonads | 3,44 |  |  |  |
| Gonadectomized | 4,03 |  |  |  |
| Difference | -0,594 | -2,23 | 1,04 | 0,46 |
| **Satisfaction** |  |  |  |  |
| Intact gonads | 3,52 |  |  |  |
| Gonadectomized | 4,22 |  |  |  |
| Difference | -0,71 | -2,06 | 0,64 | 0,15 |
| **Pain** |  |  |  |  |
| Intact gonads | 4,16 |  |  |  |
| Gonadectomized | 4,33 |  |  |  |
| Difference | -0,17 | -2,11 | 1,77 | 0,86 |
